# Functional and Evolutionary Analyses Identify Proteolysis as a General Mechanism for NLRP1 Inflammasome Activation

**DOI:** 10.1371/journal.ppat.1006052

**Published:** 2016-12-07

**Authors:** Joseph Chavarría-Smith, Patrick S. Mitchell, Alvin M. Ho, Matthew D. Daugherty, Russell E. Vance

**Affiliations:** 1 Division of Immunology & Pathogenesis, Department of Molecular & Cell Biology, and Cancer Research Laboratory, University of California, Berkeley, California, United States of America; 2 Molecular Biology Section, Division of Biological Sciences, University of California, San Diego, La Jolla, California, United States of America; 3 Howard Hughes Medical Institute, University of California, Berkeley, California, United States of America; Portland VA Medical Center, Oregon Health and Science University, UNITED STATES

## Abstract

Inflammasomes are cytosolic multi-protein complexes that initiate immune responses to infection by recruiting and activating the Caspase-1 protease. Human NLRP1 was the first protein shown to form an inflammasome, but its physiological mechanism of activation remains unknown. Recently, specific variants of mouse and rat NLRP1 were found to be activated upon N-terminal cleavage by the anthrax lethal factor protease. However, agonists for other NLRP1 variants, including human NLRP1, are not known, and it remains unclear if they are also activated by proteolysis. Here we demonstrate that two mouse NLRP1 paralogs (NLRP1A^B6^ and NLRP1B^B6^) are also activated by N-terminal proteolytic cleavage. We also demonstrate that proteolysis within a specific N-terminal linker region is sufficient to activate human NLRP1. Evolutionary analysis of primate NLRP1 shows the linker/cleavage region has evolved under positive selection, indicative of pathogen-induced selective pressure. Collectively, these results identify proteolysis as a general mechanism of NLRP1 inflammasome activation that appears to be contributing to the rapid evolution of NLRP1 in rodents and primates.

## Author Summary

Hosts and their pathogens often engage in evolutionary ‘arms races’, iterative cycles of adaptation, in which each opponent evolves strategies to overcome the other. For example, the anthrax bacterium overcomes the host immune response by producing lethal factor, a proteolytic enzyme that specifically cleaves and inactivates host immunity proteins called MAP kinases. Rodents counteract this strategy by producing a sensor protein called NLRP1 that is cleaved by anthrax lethal factor. Upon cleavage, NLRP1 activates a potent anti-bacterial immune response that compensates for the loss of the MAP kinase response. Humans also produce NLRP1, but human NLRP1 is neither cleaved nor activated by lethal factor. Thus, the mechanism of human NLRP1 activation and its function in immunity remains unknown. In our study, we show that human NLRP1, like rodent NLRP1, can be activated by proteolytic cleavage. Interestingly, evolutionary analysis supports the hypothesis that primate NLRP1 is rapidly evolving to be cleaved by (and thereby detect) pathogen-encoded proteases. Our results elucidate a general mechanism for NLRP1 activation and suggest that host immunity proteins may evolve toward recognition by bacterial proteases to engage in evolutionary arms races with pathogens.

## Introduction

Mammals have evolved multiple mechanisms to detect microbes in order to initiate immune responses during infection. While both harmless and pathogenic microbes are detected, pathogens generally induce robust responses sufficient to mediate their elimination, whereas commensals trigger milder responses that do not generally produce immunopathology. One family of pattern recognition receptors that can discriminate between pathogens and commensals is the nucleotide-binding domain (NBD) and leucine-rich repeat (LRR) containing (NLR) protein family [1–4]. NLRs are cytosolic proteins that can be activated upon pathogen access to the host cell cytosol [5]. Pathogens employ a variety of virulence factors, such as toxins and secretion systems, to access the cytosol, resulting in NLR activation [6]. By contrast, commensals do not generally encode these virulence factors. Upon activation, several NLRs have been shown to form a scaffold, termed an inflammasome, which recruits and activates the Caspase-1 protease (CASP1) [7]. Active CASP1 is required for the cleavage and release of the cytokines IL-1β and IL-18, and also initiates a lytic and inflammatory cell death known as pyroptosis.

The molecular mechanisms by which different NLRs are activated in response to pathogen stimulation are not completely understood. In one well-characterized mechanism of NLR activation, members of the NAIP subfamily of NLRs have been shown to bind directly to specific bacterial ligands such as flagellin [8–10]. Upon ligand binding, NAIPs co-associate with a different NLR member, NLRC4, to form an inflammasome complex that recruits and activates CASP1 and ASC. However, most NLRs do not appear to utilize the simple receptor-ligand activation mechanism utilized by NAIPs. For example, the NLRP3 inflammasome appears to respond to potassium efflux [11], but the underlying molecular basis for this response remains unknown.

Mouse NLRP1B is another NLR that does not appear to be activated by a receptor-ligand type mechanism. Instead, NLRP1B variants from certain inbred mouse strains, e.g., BALB/c and 129, can be activated by the lethal factor (LF) protease that is produced and secreted by *Bacillus anthracis*, the causative agent of anthrax [12]. Together with protective antigen (PA), LF forms a bipartite toxin, Lethal Toxin (LeTx). The role of PA is to form a translocation channel that delivers LF into the host cell cytosol, where LF hampers the host immune response by cleaving and inactivating most MAP kinase kinases [13,14]. In addition to cleavage of MAPKKs, which appears to promote anthrax virulence, LF also directly cleaves NLRP1B proximal to its N-terminus [15], which is both necessary and sufficient [16] for NLRP1B inflammasome formation and CASP1 activation. Activation of NLRP1B-dependent inflammasome responses appears to contribute to host defense via a mechanism requiring IL-1β and neutrophils [17,18]. Together, these results suggest NLRP1B can function as a sensor of bacterial proteases, similar to other immune responses that are specifically activated by virulence factors [19–21].

Interestingly, other members of the NLRP1 gene family do not appear to be activated by LF. NLRP1B is highly polymorphic in mice [12], and only two of five identified alleles have been shown to respond to LF. The allele found in C57BL/6 (B6) mice fails to respond to LF, but it remains unclear if this is because NLRP1B^B6^ is not cleaved by LF, or because NLRP1B^B6^ has lost inflammasome functionality. In addition, it is not clear what stimuli might activate NLRP1A, the other known functional murine NLRP1 paralog. A previous study identified a mouse carrying a missense gain-of-function mutation in NLRP1A (Q593P) that exhibits spontaneously active inflammasome responses [22], but the mechanism of wild-type NLRP1A activation is unclear. Recently, several groups have provided evidence that NLRP1B in mice and NLRP1 in rats can respond to *Toxoplasma gondii* infections [23–25]. However, the mechanism by which *T*. *gondii* activates NLRP1 is unknown. Interestingly, it has also been shown that NLRP1 can function as a metabolic sensor that is activated by reduced intracellular levels of ATP [26,27]. Thus, it remains unclear whether proteolysis represents a unique activation mechanism limited to certain rodent NLRP1 isoforms, or is instead a general mechanism that governs NLRP1 activation in diverse species.

The mechanism of activation of the human ortholog of NLRP1 is controversial and has been suggested to be distinct from that of mouse NLRP1B. Human NLRP1 was the first NLR shown to assemble into a multi-protein inflammasome complex [7], but in that study, NLRP1 was activated spontaneously in cellular lysates. Thus, the mechanism by which NLRP1 is activated in response to a specific stimulus was not addressed. Similar to mouse NLRP1B^B6^, human NLRP1 is not responsive to anthrax LF [28]. One study observed that muramyl dipeptide (MDP), a fragment of peptidoglycan found in bacterial cell walls, stimulated the oligomerization of NLRP1 in a cell-free system [29]. However, other studies raise doubts that MDP is a specific agonist for NLRP1 [30–33]. In particular, it has been difficult to dissociate a direct role for MDP in NLRP1 activation from the known ability of MDP to prime inflammasome expression via the NOD2 sensor. Importantly, genetic data demonstrating a requirement for NLRP1 in the inflammasome response to MDP is currently lacking.

The overall architecture of human and rodent NLRP1 proteins is similar, including conservation of a four domain module, NBD-LRR-FIIND-CARD, which comprises the majority of the protein. One important structural difference between human NLRP1 and rodent NLRP1 isoforms is the presence of an N-terminal Pyrin domain (PYD) in human NLRP1 that is absent from rodent NLRP1 isoforms. In other NLRs, the PYD plays an essential function in recruiting CASP1 via a PYD/CARD-containing adaptor protein called ASC. Thus, it was originally unclear whether N-terminal cleavage would activate human NLRP1, since this cleavage would disrupt or remove the PYD. However, all NLRP1 proteins also contain a C-terminal Caspase Activation and Recruitment Domain (CARD). Although the initial description of the inflammasome suggested that the CARD was insufficient to bind CASP1 directly [7], more recent data suggest that the CARD rather than the PYD is the domain in human NLRP1 that recruits and activates CASP1 [29,34,35]. Interestingly, mutations in the PYD of human NLRP1 have recently been shown to lower the threshold for spontaneous NLRP1 activation by destabilizing the PYD [36], suggesting that the PYD may play a role in maintaining NLRP1 in its inactive state. Given the mechanism of activation of mouse NLRP1B inflammasome, these findings raise the possibility that human NLRP1 could be activated by proteolytic cleavage resulting in the removal of an auto-inhibitory PYD.

In this study, we address directly whether proteolytic cleavage is a general mechanism for NLRP1 activation in rodents and humans. We took advantage of our ability to reconstitute NLRP1 inflammasomes individually in heterologous cells that lack endogenous inflammasomes to determine whether N-terminal cleavage is sufficient for NLRP1 activation. Inflammasome reconstitution avoids confounding problems of interpretation that have arisen when using cells such as macrophages, which can form multiple inflammasomes. Consistent with previous observations, we find that LF cleaves and activates only some alleles of mouse NLRP1B. But interestingly, we observed that induced N-terminal proteolysis of the B6 isoforms of rodent NLRP1B and NLRP1A is sufficient to cause inflammasome activation. Moreover, consistent with a general role for proteolytic activation, the human NLRP1 ortholog could also be activated via direct proteolysis in a specific N-terminal linker region between the PYD and NBD domains. Evolutionary analysis demonstrates that this linker region is evolving rapidly under positive selection, suggestive of pathogen-driven evolution to diversify to detect novel protease virulence factors. Taken together, our results provide a plausible and general molecular mechanism for activation of NLRP1 and suggest that host immunity proteins may evolve toward recognition by bacterial proteases to engage in evolutionary arms races with pathogens.

## Results

### Cleavage and activation of the B6 NLRP1B isoform

**Fig 1:**
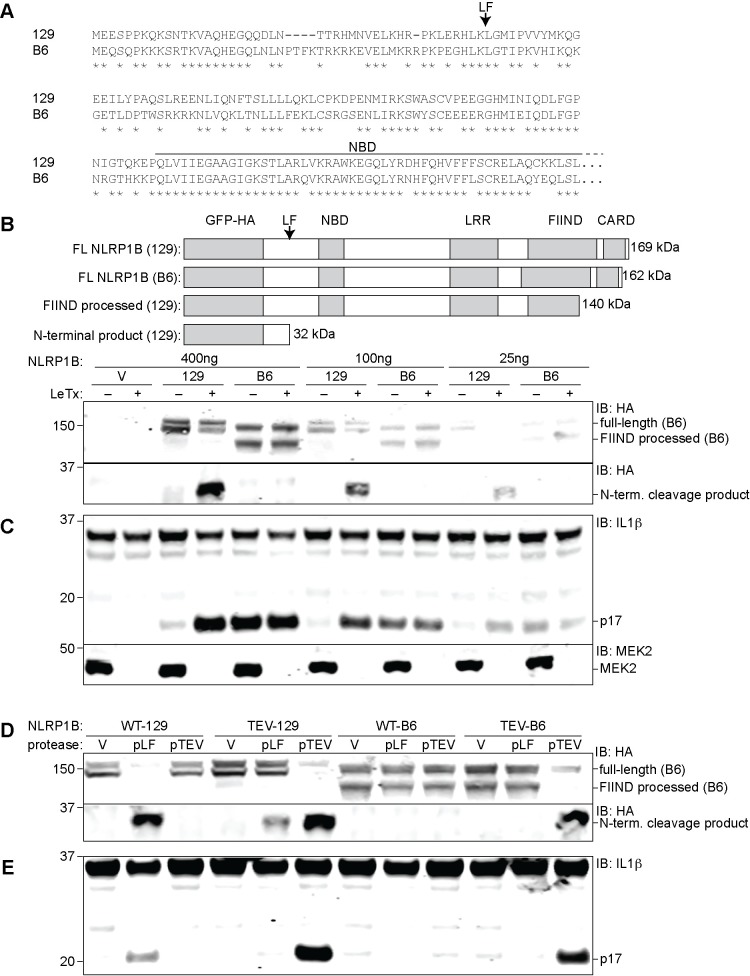
The B6 variant of NLRP1B is not cleaved by LF but can form an inflammasome in response to proteolysis. (**A**) The amino acid sequence of the first 244 residues of NLRP1B^B6^ was aligned to the homologous sequence of NLRP1B^129^. The arrow above the alignment indicates the LF-cleavage site in NLRP1B^129^. Asterisks indicate sites of amino acid identity. (**B**) For detection of NLRP1B expression, 293T cells were transfected with the indicated amounts of and empty vector (V) or plasmids encoding GFP-HA-NLRP1B^129^ or GFP-HA-NLRP1B^B6^ (construct schematics and the predicted molecular weight of each protein is depicted in the upper panel). Cells were treated overnight with anthrax lethal toxin protein (LeTx, 1μg/ml) 24h post-transfection, and then lysates were analyzed by immunoblotting (IB) with the indicated antibodies. For NLRP1 expression and cleavage, CASP1 and IL1B were omitted to prevent cell death and resulting apparent differences of expression. For blots probed with anti-HA (NLRP1B), the lysates were not boiled prior to loading to prevent aggregation and smearing of full-length and FIIND-processed NLRP1B on the immunoblot. To visualize the N-terminal processed form of NLRP1B, the lysates were boiled and resolved on a separate gel. (**C**) 293T Cells were transfected with the same amount of titrated NLRP1B encoding plasmid and treated as in B, but transfections also included plasmids encoding mouse CASP1 (200ng), IL-1β (200ng) and 200ng of empty vector. (**D**) For detection of NLRP1B expression, 293T cells were transfected for 36h with 250ng of plasmids encoding WT 129 or B6 NLRP1B or mutants engineered to express the TEV-protease site. Each plasmid was co-transfected with either 100ng of empty vector (V) or plasmids encoding TEV-protease (pTEV) or lethal factor protease (pLF), supplemented with 300ng empty vector (pMSCV). Cells were not treated with LeTx and lysates were analyzed by immunoblotting as in (B). (**E**) Cells were treated as in C, but with 8ng of plasmids encoding NLRP1B, along with 200ng of plasmids encoding mCASP-1 and mIL-1β, and 200ng empty vector to normalize plasmid quantities. Lower quantities of NLRP1B were transfected as compared to panel D so to avoid spontaneous NLPR1B activation. For panels B-E, data shown are representative of at least three similar experiments.

NLRP1B is highly polymorphic in mice, and five different allelic variants have been identified [12]. Of these five variants, only two respond to LF to form an inflammasome to promote pyroptosis. Although the NLRP1B variant from B6 mice (NLRP1B^B6^) is expressed [37], it is not responsive to lethal toxin (LeTx), and indeed, it is unclear if NLRP1B^B6^ can even form an inflammasome. Alignment of the amino acid sequence of NLRP1B^B6^ to the LF-responsive NLRP1B^129^ isoform reveals considerable variation between alleles, with only 84.6% amino acid identity. Interestingly, most of these polymorphisms occur in the N-terminal portion of the protein (Fig 1A), suggesting they may affect N-terminal proteolysis by LF. We therefore first investigated if LF cleaves the B6 isoform of NLRP1B.

In our studies, we decided to reconstitute a functional NLRP1 inflammasome response in transfected HEK 293T cells. Reconstituted systems have previously been used to study NLRP1 [16,26,35,38] and provide several advantages: (1) 293T cells lack other inflammasomes; thus, we avoid the confounding effects other inflammasomes have had on prior studies of endogenous NLRP1, e.g., in THP-1 cells; (2) A reconstituted system allows us to easily express modified NLRP1 alleles (e.g., point mutants, GFP fusions, epitope-tagged alleles, etc.) that are critical to our studies; (3) A reconstituted system provides a uniform genetic background for testing various NLRP1 alleles, and thus allows for direct comparisons across alleles; (4) By titrating the amount of plasmid used in transfections, our reconstituted system also permits control over the levels of inflammasome gene expression. This latter point is critical because overexpression of NLRs tends to lead to their spontaneous activation. This can be useful to establish that a given NLRP1 protein is potentially functional (i.e., properly folding), especially when a physiological stimulus to activate a given NLRP1 isoform is unknown. However, for our studies of NLRP1 activation by proteolysis, it is also possible to express NLRP1 at lower levels (below the threshold for spontaneous activation), as we describe below.

Thus, 293T cells were transfected with expression plasmids encoding either GFP-HA-NLRP1B^129^ or GFP-HA-NLRP1B^B6^, along with expression plasmids encoding CASP1 and IL-1β to reconstitute a functional inflammasome. All NLRP1 proteins contain a FIIND (Function-to-find Domain), which is an auto-processing domain that spontaneously cleaves itself to generate two polypeptides that are believed to remain non-covalently associated in the mature NLRP1 protein [34,35,39]. For reasons that remain unclear, FIIND auto-processing is essential for the function of both rodent and human NLRP1 [34,35]. As expected, we observed that transfected NLRP1B^B6^ appears as two bands on SDS PAGE, implying that the protein is properly folded and that FIIND auto-processing can occur. Surprisingly, expression of NLRP1B^B6^ promoted significant amounts of IL-1β processing even in the absence of LF. This suggests that, despite numerous polymorphisms, NLRP1B^B6^ is able to form an inflammasome when over-expressed (Fig 1B). However, the large amount of cleaved IL-1β observed in cells expressing NLRP1B^B6^ was unexpected, and was much greater than the basal activity of the 129 allele, despite comparable expression and FIIND processing for both isoforms. Importantly, LeTx treatment did not further increase the amount of cleaved IL-1β (p17) in cells expressing the B6 allele, though LeTx did stimulate IL-1β processing by cells expressing NLRP1B^129^ (Fig 1B). Cleavage of the known LeTx target MAP kinase MEK2 served as an additional positive control for LeTx addition (Fig 1C). We did not observe LeTx-dependent cleavage of the B6 isoform, but did observe the expected cleavage of the 129 isoform. In order to reduce the levels of spontaneous activation seen in cells expressing NLRP1B^B6^, we titrated the amount of expression plasmid used in the transfections. We observed a dose-dependent decrease in the amount of spontaneous IL-1β processing under these conditions (Fig 1B), but were still unable to reveal LeTx-induced inflammasome activity. We conclude that NLRP1B^B6^ is capable of forming an inflammasome, but it is not cleaved nor activated by LF, and exhibits weaker intrinsic auto-inhibition that can be overcome under conditions of overexpression.

While NLRP1B^B6^ is neither cleaved nor activated by LF, we hypothesized that it might nevertheless be cleaved and activated by unknown proteases with specificities different than the LF protease. Following a strategy we used previously with NLRP1B^129^ [16], we tested this hypothesis by engineering a tobacco etch virus (TEV) protease cleavage-site in the NLRP1B^B6^ isoform. The TEV site was inserted in NLRP1B^B6^ at a position corresponding to the site at which NLRP1B^129^ is cleaved by LF. We then transfected cells with reduced amounts of expression plasmids encoding the NLRP1B^B6^-TEV allele (insufficient to produce spontaneous inflammasome formation) along with plasmids encoding TEV or LF protease, and assessed inflammasome formation via detection of cleaved IL-1β by immunoblot. Interestingly, TEV protease, but not LF protease, was able to induce significant IL-1β processing in cells expressing the NLRP1B^B6^-TEV isoform (Fig 1D and Fig 1E). The TEV-dependent activation of NLRP1B^B6^-TEV was comparable in magnitude to that seen with NLRP1B^129^-TEV, or to the LF-dependent activation of wild-type NLRP1B^129^. Consistent with the lack of cleavage observed upon LeTx treatment (Fig 1B), the WT B6 isoform was neither cleaved nor responsive to LF over-expression (Fig 1C). Similarly, as a further specificity control, over-expression of Dengue virus NS2B/NS3 protease, which is known to target host cell substrates [40] had no effect on NLRP1B^129^ activation (S1 Fig). The Dengue protease was clearly active as it underwent auto-proteolytic cleavage required for its activity. These results are consistent with a model in which NLRP1B activation occurs through specific protease recognition and cleavage.

We next assessed why NLRP1B^B6^ is not cleaved by LF. NLRP1B^B6^ exhibits three amino acid substitutions in the region corresponding to the LF-cleavage site in NLRP1B^129^ (Figs 1A and S2A), and we hypothesized that these polymorphisms might explain the differential LF proteolysis of the two isoforms. We mutated these three residues in the 129 allele to match the ones found in the B6 allele, and then tested the sensitivity of the resulting proteins to LF cleavage. 129/B6-A (L39P/R41G) and 129/B6-B (M47T) exhibited little to no reduction in LF-dependent cleavage and inflammasome responsiveness (S2B Fig). Combining the two sets of mutations to generate 129/B6-C resulted in a protein with a partially reduced level of LF-cleavage. A previous paper examining cleavage of a polypeptide encoding the first 118 amino acids of NLRP1B^129^ indicated that K38 and K44 were necessary for susceptibility to cleavage by LF [41], but we found that mutating both residues simultaneously in the context of the full-length protein did not affect sensitivity of NLRP1B^129^ to cleavage (S2C Fig). This indicates that K38 and K44 residues are incomplete determinants of LF sensitivity. Our results suggest that in addition to cleavage site polymorphisms, polymorphisms outside the immediate vicinity of the cleavage site might also contribute to differential proteolysis of the two isoforms. This conclusion is consistent with what is known about the mechanism of MAPKK recognition by LF (see Discussion).

### Cleavage and activation of mouse NLRP1A

**Fig 2:**
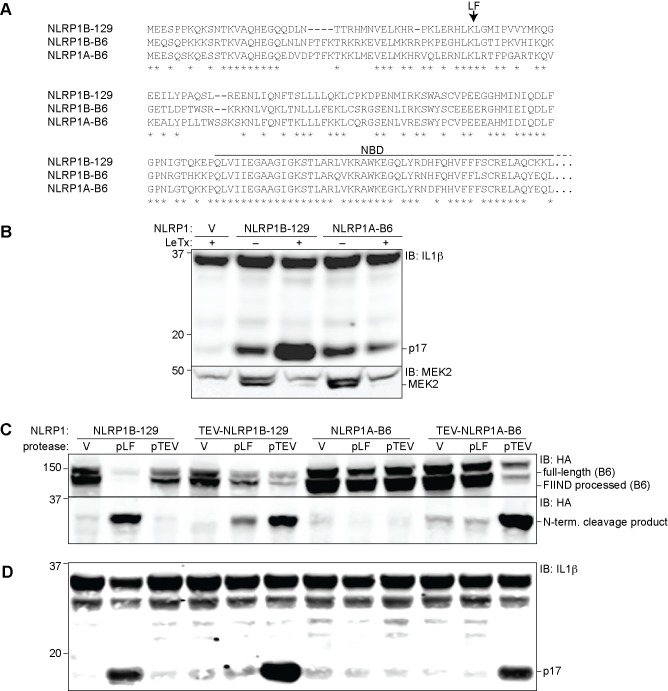
NLRP1A is activated by N-terminal proteolysis. (**A**) The amino acid sequence of the first 244 residues of NLRP1A^B6^ was aligned to NLRP1B^129^ and NLRP1B^B6^. The arrow above the alignment indicates the LF-cleavage site in NLRP1B^129^. (**B**) 293T cells were transfected for 36h with 200ng empty vector (V) or plasmids pcDNA3.1-HA-NLRP1B^129^ or -NLRP1A^B6^-MYC (CMV promoter) along with 200ng mCASP1 and 200ng mIL-1β expression vectors. Cells were treated overnight with anthrax lethal toxin (LeTx, 1μg/ml) 24h post-transfection, and then lysates were analyzed by immunoblotting (IB) with the indicated antibodies. (**C**) 293T cells were transfected for 36h with plasmids encoding 400ng GFP-HA-NLRP1B^129^ or GFP-HA-NLRP1A^B6^ (under the control of the LTR promoter in pMSCV) or with mutants engineered to contain an N-terminal TEV protease site. Cells were also co-transfected with 100ng empty vector (V) or plasmids encoding TEV-protease (pTEV) or lethal factor protease (pLF), plus 300ng empty pMSCV. (**D**) To assess IL-1β cleavage, cells were transfected as in C, but with 8ng of GFP-HA-NLRP1B and co-transfected with vectors encoding 200ng mCASP1 and 200ng mIL-1β. For panels B-D, data shown are representative of at least three similar experiments.

B6 macrophages also express a transcript for NLRP1A [12,37], but no exogenous stimulus has been identified that can activate NLRP1A. Since B6 macrophages do not respond to LF, it has been presumed that NLRP1A^B6^ is not activated by LF. Comparison of the primary amino acid sequences of NLRP1A^B6^ versus NLRP1B^B6^ again reveals numerous N-terminal polymorphisms (Fig 2A). However, it remained unclear whether NLRP1A^B6^ is cleaved by LF and whether NLRP1A^B6^ could form a functional inflammasome in response to proteolysis.

We therefore generated a construct to express the reference sequence for NLRP1A^B6^ found in NCBI (NM_001004142.2). We first tested if high levels of expression of NLRP1A^B6^ are sufficient to produce a functional inflammasome in the 293T reconstituted system described above. Overexpression of NLRP1A^B6^ under a strong (CMV) promoter indeed led to substantial IL-1β processing, comparable to the amount induced by overexpression of NLRP1B^129^ (Fig 2B). Consistent with the lack of responsiveness of B6 macrophages to LeTx, LeTx did not enhance the amount of IL-1β processing in cells expressing NLRP1A^B6^, whereas cells expressing NLRP1B^129^ were responsive to LeTx as expected. To test directly whether LF can cleave NLRP1A^B6^, we fused GFP-HA to the N-terminus of NLRP1A as described above for NLRP1B. Co-expression of GFP-HA-NLRP1A^B6^ and LF did not result in detectable N-terminal cleavage, which is robustly seen with NLRP1B^129^ (Fig 2C).

In order to test whether proteolysis is sufficient to activate NLRP1A^B6^, we engineered a TEV-protease site into NLRP1A at a position corresponding to the site of LF cleavage in NLRP1B^129^ (Fig 2A). In these experiments, we transfected less NLRP1 plasmid and used a plasmid encoding a weaker (LTR) promoter to reduce spontaneous activation. Interestingly, specific proteolysis of NLRP1A^B6^-TEV induced significant IL-1β processing, comparable to the cleavage-induced IL-1β processing observed with wild-type NLRP1B or NLRP1B^129^-TEV (Fig 2C). Importantly, this TEV-induced activation correlated with N-terminal cleavage of NLRP1A^B6^, and was not observed with NLRP1 forms that lack the TEV-protease site.

### Cleavage and activation of human NLRP1

The LF cleavage sites identified in rodent NLRP1 variants do not appear to be conserved in human NLRP1 (hNLRP1), and treatment of human macrophages with LeTx does not lead to inflammasome activation [28]. In addition, human and primate NLRP1 variants contain an N-terminal Pyrin domain (PYD), which is shared with numerous mammals, but has been lost in the recently duplicated rodent NLRP1 variants (S3A and S3B Fig). The PYD is connected to the NBD via an inter-domain linker >100 amino acids in length that has no predicted secondary structure (S4 Fig). Although hNLRP1 is not cleaved by LF, both hNLRP1 and rodent NLRP1 contain a predicted unstructured region directly N-terminal to the NBD. A hypothetical cleavage event within this region of hNLRP1 would liberate a C-terminal fragment containing the NBD-LRR-FIIND-CARD domains that would resemble the active cleaved form of rodent NLRP1. We therefore hypothesized that the unstructured inter-domain linker region in hNLRP1 might be sensitive to proteases and that proteolysis could lead to NLRP1 activation.

**Fig 3:**
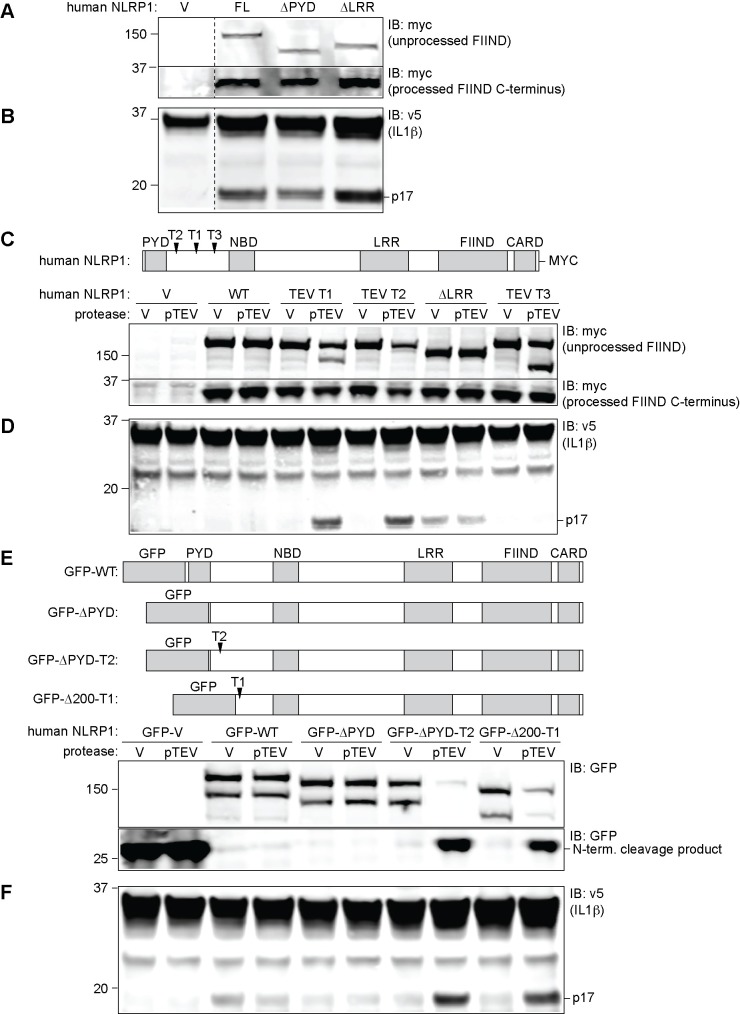
Human NLRP1 is activated by proteolysis that removes the N-terminal PYD. (**A**) For detection of NLRP1 expression, 293T cells were co-transfected for 24h with 100ng human full-length pcDNA3.1-NLRP1-MYC (CMV promoter) (FL), or with constructs in which the leucine rich repeat or pyrin domains had been deleted (ΔLRR, ΔPYD). Cells were also co-transfected with 500ng empty pcDNA3.1 vectors and with plasmids encoding human ASC (1ng), hCASP1 (1ng), and IL-1β–V5 (200ng). After 36h, cells were analyzed by immunoblot (IB) using an antibody against MYC. (**B**) Cells were transfected as in A, but to avoid spontaneous NLRP1 activation, the amount of hNLRP1 plasmid was reduced to 10ng and adjusted with 90ng of empty vector. Lysates were blotted with an anti-V5 antibody. (**C**) Cleavage sites for TEV protease were introduced into pcDNA3.1-hNLRP1-MYC at the indicated positions (see S4 Fig for details) to generate TEV T1, TEV T2, and TEV T3 expression constructs. 293T cells were co-transfected with 10g of these NLRP1 constructs along with 50ng of a vector encoding TEV-protease (pTEV) or a control empty vector (V), and all supplemented with 200ng empty pcDNA3.1. After 24h, lysates were analyzed by immunoblot using anti-Myc antibody to detect full-length NLRP1, the FIIND-processed (30kDa) C-terminal product, or the products that result from TEV cleavage (144kDa for T1, 155kDa for T2, and 133kDa for T3). (**D**) 293T cells were co-transfected with 5ng of NLRP1 constructs along with 50ng of vector encoding TEV-protease (pTEV) or a control empty vector (V), and plasmids encoding human ASC (1ng), CASP1 (1ng), and IL-1β-V5 (200ng), supplemented with 500ng empty pcDNA3.1. After 24h, lysates were analyzed by immunoblot using anti-V5 antibody to detect CASP1-dependent processing of IL-1β into the mature p17 fragment. (**E**) A panel of constructs for expressing full-length or truncated GFP-fused human NLRP1 in pMSCV (LTR promoter) was generated as depicted. The TEV T1 and T2 sites were introduced into two of the constructs as indicated (also see S4 Fig). 293T cells were transfected with 100ng these constructs along with 50ng empty vector or a plasmid encoding the TEV protease (pTEV). Cell lysates were analyzed by immunoblot (IB) with anti-GFP. (**F**) 293T cells were transfected for 24h with 12.5ng plasmids encoding full-length or truncated GFP-fused human NLRP1 in pMSCV (LTR promoter), along with plasmids encoding human ASC (1ng), CASP1 (1ng), V5-tagged IL-1β (200ng) and TEV protease (50ng, pTEV). After 24h, lysates were analyzed by immunoblot using anti-V5 antibody to detect CASP1-dependent processing of IL-1β into the mature p17 fragment. For all panels, data shown are representative of at least three similar experiments.

One difficulty with the hypothesis that hNLRP1 is activated by proteolytic cleavage within the inter-domain linker region is that such a cleavage event would result in removal of the N-terminal PYD. An initial report suggested that the PYD was necessary for ASC and CASP1 co-recruitment by hNLRP1 [7], but recent reports suggest that the C-terminal CARD is sufficient for CASP1 activation and that the PYD fulfills an auto-inhibitory function in hNLRP1 [34,36]. We first decided to test the roles of the PYD and N-terminal cleavage in hNLRP1 activation in our reconstituted system. We assembled a human NLRP1 cDNA encoding the reference sequence isoform 1 (NM_033004.3) and expressed this cDNA in 293T cells with human CASP1, ASC, and IL-1β expression vectors to reconstitute a fully human NLRP1 inflammasome. As a positive control, we also generated an expression construct encoding a variant of hNLRP1 deleted of its LRR domain, as ΔLRR mutants of NLR proteins are typically constitutively activated [10,31,38,42]. In addition, we generated a construct lacking the N-terminal PYD to test its necessity for inflammasome function. When overexpressed at levels that promote spontaneous activation, wild-type, ΔLRR and ΔPYD hNLRP1 all induced IL-1β cleavage (Fig 3A and 3B), confirming that the PYD is dispensable for inflammasome function, and thus, proteolysis of the N-terminal PYD domain could conceivably result in an active hNLRP1 protein.

To assess whether N-terminal cleavage of hNLRP1 is sufficient to induce inflammasome activation, we engineered three different TEV-protease sites (T1-T3) into the inter-domain linker (Figs 3C and S4). We tested the ability of TEV protease to activate these NLRP1 variants, when expressed at lower levels (LTR promoter) to reduce spontaneous activation. TEV-induced cleavage at T1 and T2 resulted in significant IL-1β processing that depended on the TEV-protease sites, as TEV did not induce IL-1β processing by the wild-type version of hNLRP1 (Fig 3D). Cleavage of hNLRP1 resulted in a level of activation comparable to that seen with the constitutively active ΔLRR mutant. Interestingly, TEV-induced cleavage at T3 did not result in IL-1β processing, indicating that cleavage must occur within a specific region to induce hNLRP1 inflammasome activation.

The PYD is present in most NLRP1 orthologs, with mice and rats representing the main exceptions among mammals (S3 Fig). Our observation that the PYD is not essential for human NLRP1 activation raises the important question of why the PYD has been conserved in most mammalian NLRP1 isoforms. We hypothesized that perhaps the PYD is necessary for maintaining auto-inhibition of NLRP1, and that proteolysis might remove this auto-inhibition. To assess whether the PYD is specifically required to maintain NLRP1 auto-inhibition, we generated a series of expression constructs in which various portions of the N-terminus of hNLRP1, including the PYD, was replaced by GFP (Fig 3E). Surprisingly, an hNLRP1 mutant in which the PYD was replaced by GFP was still auto-inhibited, and was still able to be activated by proteolysis at T1 or T2 (Fig 3F). These results suggest that the PYD is neither specifically required for auto-inhibition nor for activation of hNLRP1.

### The N-terminal linker region of primate NLRP1 is evolving under positive selection

**Fig 4:**
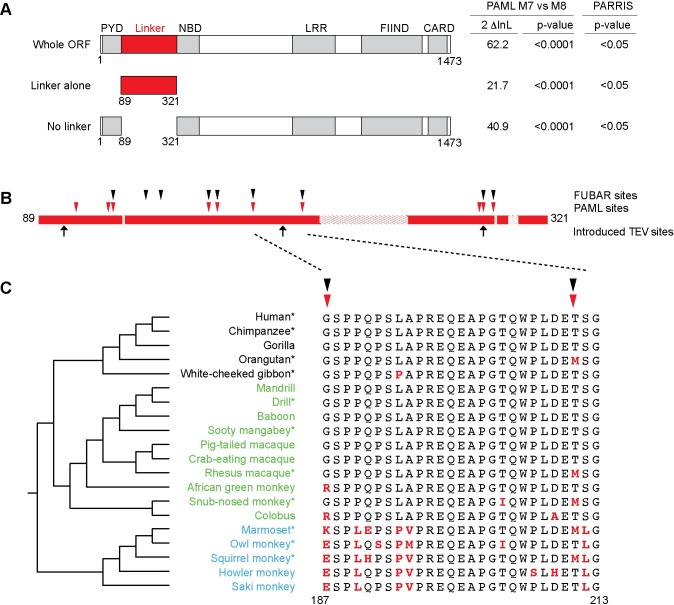
Evolutionary analysis of positive selection in primate *NLRP1* genes. (**A**) Results of PAML [46] and PARRIS [47] maximum likelihood analyses using 11 primate *NLRP1* genes, providing evidence for positive selection for the entire *NLRP1* gene, just the N-terminal linker region, or the entire ORF with the linker removed. The left two columns show two times the log likelihood difference and statistical support (p-value) comparing a model that allows (M8) or disallows (M7) positive selection in PAML. The right column shows the statistical support for a similar analysis in PARRIS. (**B**) Analyses of the N-terminal linker region of 20 primate *NLRP1* genes reveal several codons have recurrently evolved under positive selection. An expanded view of the N-terminal linker region is shown, with hatched lines indicating regions that have lineage-specific insertions or deletions. Locations of individual codons that were identified by PAML [46] and FUBAR [55] maximum likelihood methods as evolving under positive selection are marked above the schematic. Locations of TEV sites introduced in this study are marked below the schematic. (**C**) Alignment of a portion of the linker region. At left is shown a phylogenetic tree of the analyzed species with hominoids in black text, Old World monkeys in green text and New World monkeys in blue text. Species marked with asterisks were also used for the analyses shown in panel (A). Amino acids marked in bold red differ from human NLRP1.

The above data suggest that human NLRP1 could act as a sensor of bacterial proteases. Such a model predicts that the region that is cleaved by bacterial proteases for activation might need to rapidly and recurrently evolve to become an effective substrate for novel bacterial proteases. To address the plausibility of this hypothesis, we investigated the evolutionary history of primate NLRP1 with specific focus on the inter-domain linker. A previous analysis indicated that NLRP1, at a whole gene level, exhibits a strong overall signature of positive (‘diversifying’) selection [43] as measured by an excess of amino-acid altering mutations over what would be expected by the neutral theory of molecular evolution [44]. Such a signature of positive selection is often observed in immune related genes as a consequence of an evolutionary ‘arms race’ with pathogens [45]. To pinpoint the regions of NLRP1 that were diversifying under positive selection, we analyzed sequences of NLRP1 from 11 primate genomes (S1 Table) using two methods for detection of positive selection (PAML [46] and PARRIS [47]). These analyses confirmed that primate NLRP1 has a very high likelihood of having evolved under positive selection (Fig 4A; PAML M7 vs M8 p<0.0001; PARRIS p<0.05). Interestingly, a strong signature of positive selection is evident when the inter-domain linker region between the PYD and the NBD (residues 81–321 in human NLRP1) is analyzed alone (without consideration of the rest of NLRP1) indicating that residues in this region have recurrently evolved under positive selection (specific codons detailed in S2 Table). Removal of this region from the analyses does not eliminate the signature of positive selection (Fig 4A), suggesting that additional regions of the protein (e.g., the LRRs) have also evolved under positive selection (specific codons detailed in S3 Table). These additional regions may also contribute to protease recognition, or alternatively, may be evolving in response to distinct selective pressures (e.g., pathogen-encoded inhibitors). Codon-based analyses of the N-terminal linker region from 20 divergent primate NLRP1 genes identified several individual codons that exhibit statistically (posterior probability >0.9) significant signatures of positive selection (Fig 4B and 4C). These data strongly suggest that the inter-domain linker region plays an important role in NLRP1 function and that it has undergone repeated pathogen-driven selection to diversify in amino acid sequence. Consistent with these data in primates, we see evidence for positive selection acting on the N-terminus of mouse NLRP1 alleles based on a pairwise comparison of *Nlrp1a* and *Nlrp1b* (S3C Fig). These results, combined with the importance of this region in protease-mediated NLRP1 activation in both rodents and primates, are consistent with a model in which the linker region is evolving rapidly to acquire the ability to be cleaved by—and thus detect—pathogen-encoded proteases of differing sequence specificities.

### Activation of hNLRP1 does not require an intact ATP-binding Walker A motif

**Fig 5:**
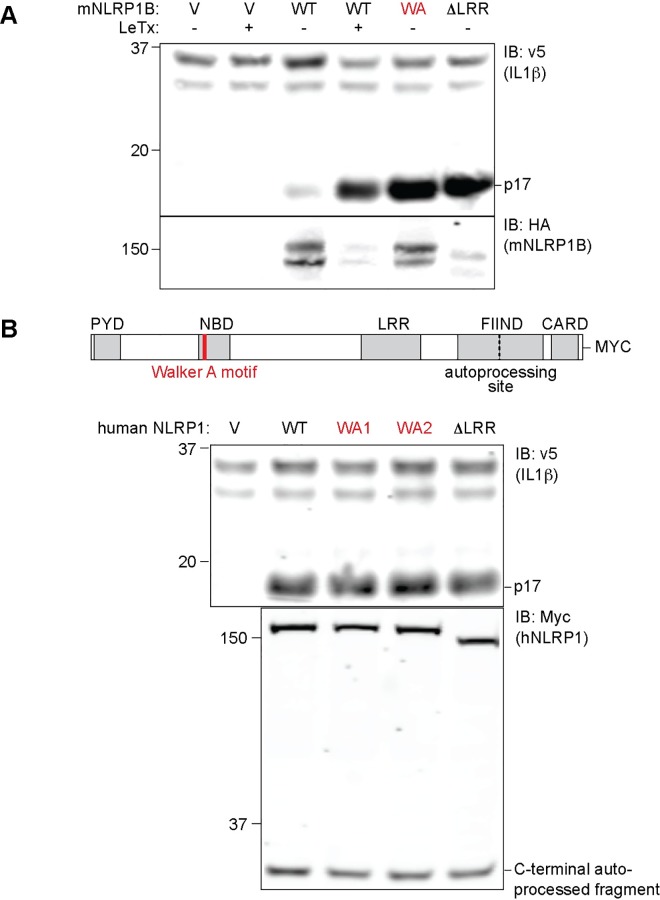
Human NLRP1 does not require a Walker A motif for inflammasome assembly. (**A**) 293T cells were transfected with 100ng of CMSCV empty vector (V) or plasmids encoding mouse wild type HA-NLRP1B^129^ (WT) or mutant HA-NLRP1B^129^ either containing the K137R mutation in the Walker A (WA) ATP-binding site or the constitutively active ΔLRR mutant. The cells were co-transfected with vectors encoding mouse CASP1 and IL-1β. Cells expressing mouse NLRP1B were treated with LeTx for 16h where indicated. Non-boiled lysates were subject to immunoblot (IB) with anti-HA antibody, whereas boiled lysates were subject to immunoblot with anti-IL-1β to detect CASP1-dependent processing of IL-1β into the mature p17 fragment. (**B**) 239T cells were transfected with 100ng empty vector (V) or plasmids encoding hNLRP1-MYC wild-type (WT), Walker A mutant 1 K340A (WA1), Walker A mutant 2 K340R (WA2) or ΔLRR expression plasmids. The transfection complexes also contained 200ng mCASP, 200ng mIL-1β and 1ng hASC expression vectors. For both A and B, data shown are representative of at least three similar experiments.

Since the above data imply that rodent and human NLRP1 can be activated by a conserved mechanism involving N-terminal proteolysis, we decided to evaluate other reported differences between human and mouse NLRP1. ATP binding to human NLRP1 has been reported to be required for inflammasome oligomerization and CASP1 activation [29]. By contrast, a requirement for ATP binding is not exhibited by mouse NLRP1B. Instead, prevention of ATP binding by mutation of the conserved Walker A motif appears to lead to a constitutively active mouse NLRP1B [26] (Fig 5A). We decided to test the effect of mutating the Walker A motif (K340A and K340R) in human NLRP1. Surprisingly, but consistent with observations with mouse NLRP1B, Walker A mutations did not abrogate the ability of hNLRP1 to promote IL-1β processing mediated by hNLRP1 (Fig 5B). This further indicates that fundamental aspects of the mechanism of NLRP1 activation are conserved between rodents and humans.

## Discussion

The recent finding that the lethal factor (LF) protease activates NLRP1B^129^ via direct proteolytic cleavage [16,41] has raised the question whether proteolysis is a general mechanism of NLRP1 activation. Although multiple allelic and paralogous variants of NLRP1 have been described, no clear agonist for most of these variants has been discovered, and for some variants it remains unclear whether they exhibit inflammasome function at all. Human NLRP1 has been proposed to be activated by muramyl dipeptide (MDP), a fragment of bacterial peptidoglycan [29], suggesting that the mechanisms of hNLRP1 and rodent NLRP1 activation might be entirely distinct. In contrast, we show here that proteolysis can act as a common activator of diverse NLRP1 variants from mice and humans.

We first evaluated the sensitivity of NLRP1A and NLRP1B of B6 mice to cleavage by the LF protease, and found that neither NLRP1A^B6^ nor NLRP1B^B6^ were cleaved by LF. This result directly accounts for the prior observation that B6 macrophages do not form an inflammasome when they are treated with lethal toxin (LeTx) [12]. However, the reason why neither of these paralogs are cleaved by LF is harder to discern. We found that conversion of the cleavage site in NLRP1B^129^ to the sequence present in NLRP1B^B6^ only had a modest effect on cleavage of NLRP1B^129^. These data suggest that other regions of NLRP1B contribute to the specificity of LF for NLRP1B^129^ versus NLRP1B^B6^. This hypothesis is consistent with the way in which LF recognizes its MAPKK substrates [13], which is dictated not only by the sequence of the cleavage-site itself but also by a C-terminal region termed the LFIR [48]. Similarly, we suspect that another domain of NLRP1B might contribute to its interaction with LF.

Despite the inability of LF to cleave NLRP1A^B6^ or NLRP1B^B6^, we were able to demonstrate that proteolytic cleavage is sufficient to activate both isoforms. Although identification of a gain-of-function mutation previously suggested that NLRP1A^B6^ can form an inflammasome, our data provide the first evidence that NLRP1B^B6^ is likewise able to activate Caspase-1 (CASP1). In fact, we unexpectedly observed that NLRP1B^B6^ exhibits high basal inflammasome activity, as compared to NLRP1B^129^, when overexpressed in 293T cells. This spontaneous activity is unlikely to be physiologically relevant since endogenous NLRP1B in macrophages is likely expressed at levels significantly below the threshold that produces spontaneous activation. Taken together our data suggest that both NLRP1A^B6^ and NLRP1B^B6^ can be activated by a conserved cleavage-dependent mechanism, but these NLRP1 paralogs appear to have evolved to respond to proteases or stimuli other than LF. Since *Bacillus anthracis* is not known to exert a major selective pressure on natural rodent populations, the loss of responsiveness of NLRP1 to LF might not incur a significant fitness cost, especially if the loss of responsiveness to LF was accompanied by the acquisition of responsiveness to a more significant pathogen. Our data demonstrate that NLRP1A^B6^ and NLRP1B^B6^ are potentially activated by proteolysis, and suggest that identification of cognate proteases will be an important future avenue for research.

Although human NLRP1 was the first NLR described to form an inflammasome [7], its proposed mechanism of activation has been controversial, and surprisingly distinct from that of mouse NLRP1B. In the initial study, it was claimed that the PYD mediates ASC and CASP1 co-recruitment [7]. However, a recent study disputed this conclusion, reporting that the PYD was dispensable for CASP1 activation or ASC binding [34]. Moreover, the observation that mutations in NLRP1 that destabilize the PYD result in spontaneous NLRP1 inflammasome activation suggest that the PYD may play a role in maintaining NLRP1 in an inhibited state [36]. Our results augment these prior findings and suggest that cleavage of the PYD releases NLRP1 from its auto-inhibited state. Unexpectedly, we were able to replace the PYD with an unrelated protein fold (GFP) without loss of NLRP1 auto-inhibition. This suggests that the PYD might mediate auto-inhibition of NLRP1 via a mechanism involving steric hindrance rather than specific intramolecular contacts with the PYD. This model might also explain why rats and mice could have evolved to lose the PYD altogether, as an unrelated N-terminal sequence appears sufficient to replace the PYD and maintain auto-inhibition.

Our results do not rule out the possibility that the PYD has additional functions beyond auto-inhibition. Indeed, recent studies have shown that some plant NLRs acquire accessory domains, termed ‘integrated decoys’, that exist primarily to serve as the target of pathogen-encoded virulence factors [20,21]. For example, some plant pathogens encode virulence factors that disrupt the function of WRKY-family transcription factors, e.g., by acetylation. In response, certain plant NLRs have apparently acquired WRKY domains, not for the purpose of mediating transcription, but instead to serve as ‘decoys’ that allow the NLR to detect WRKY disruption. Modification of the WRKY domain in the NLR by the virulence factor was shown to result in NLR activation and anti-pathogen responses. By analogy, it is conceivable that the function of the PYD in NLRP1 is to function as a decoy that detects pathogen-encoded virulence factors that target PYD-containing proteins. In such a scenario, the PYD would not need to have any intrinsic signaling function, but would merely need only to resemble a PYD sufficiently enough to be subject to pathogen modification and attack. Disruption of the PYD (by proteolysis, or perhaps by other modifications) could then lead to NLRP1 activation and initiation of host defense

Regardless of the above speculation, and despite previous indications to the contrary, our results suggest that the mechanism of human NLRP1 activation closely resembles that of rodents. Like mouse NLRP1, we find that full-length hNLRP1 auto-processes its FIIND domain, and that FIIND auto-processing appears to be required for inflammasome activity, in agreement with a previous report [34]. Our results conflict with those of the Reed group [29], which utilized a hNLRP1 variant that lacks exon 14, an exon that encodes part of the FIIND domain essential for NLRP1 activity [34]. Faustin et al. had also previously observed a requirement for the ATP-binding Walker A motif for NLRP1 oligomerization and CASP1 activation, whereas mouse NLRP1B Walker A mutants were previously found to be spontaneously active [26], a result we confirm here. Because Faustin et al had utilized a variant of NLRP1 lacking exon 14, we decided to re-evaluate the effect of the Walker A mutation in the context of the full-length human NLRP1 in cells. Consistent with what had previously been observed with mouse NLRP1, we found that an intact Walker A motif is dispensable for hNLRP1 inflammasome formation. Thus, human NLRP1 exhibits more structural and functional similarity to mouse NLRP1 than previously appreciated. Importantly, this similarity extends even to the mechanism of NLRP1 activation, as we were also able to show that N-terminal proteolysis is sufficient to activate hNLRP1, just as we observed for all mouse NLRP1 variants tested. Thus we propose that proteolysis can be a general mechanism of NLRP1 activation in diverse species, but the proteases that activate NLRP1 may differ for different NLRP1 variants. Our results do not rule out the possibility that additional conserved mechanisms for NLRP1 activation may also exist [26].

Genome-wide evolutionary analyses have previously shown that NLRP1 exhibits a strong signature of positive selection [43]. Positive selection is commonly observed in immune-related defense genes, driven by their participation in an evolutionary ‘arms race’ with pathogens [45]. Under this ‘arms race’ model, pathogens evolve mechanisms to evade or disable recognition by the immune system, which in turn counter-selects for variation in host defense genes to re-establish pathogen recognition. Many examples of this form of co-evolution have been observed with viruses and host defense pathways [45]. Indeed, we were previously able to identify signatures of positive selection in a different sub-family of NLRs called the NAIPs [49]. Interestingly, the region of NAIPs undergoing positive selection was the same region implicated in the specific detection of bacterial ligands. In our analysis of NLRP1 evolution in primates, we were able to extend previous analyses by identifying specific amino acid positions that showed strong evidence of positive selection. Many of the positively selected codon positions we identified mapped to the inter-domain region between the PYD and the NBD. Remarkably, this linker region is the same region in which we found that proteolysis can activate human NLRP1. Secondary structure predictions also indicate that this region is a large unstructured coil that spans more than 100 residues past the PYD (S2 Fig), facilitating protease sensitivity. An unstructured linker may also be relatively evolutionarily unconstrained and could therefore be free to alter amino acid sequences to acquire the ability to be cleaved by novel proteases. Considering the high level of sequence diversification in this linker region within primates and rodents, it is not surprising that the same LF protease that activates mouse NLRP1B does not activate other NLRP1 proteins. Rather, we expect that each NLRP1 linker has evolved to be recognized and cleaved by a unique protease (or set of proteases) from pathogens that are specific to a particular host. Consistent with the idea that mammalian hosts can detect the proteolytic activity of pathogen-encoded virulence factors, it was recently reported that the SpeB protease of group A *Streptococcus* can be detected through direct cleavage of IL-1β [50].

Taken together, our results definitively establish direct N-terminal proteolysis as a sufficient signal to activate all NLRP1 variants we tested, including mouse NLRP1A^B6^, mouse NLRP1B^B6^, and human NLRP1. It will obviously be of great interest to identify proteases, other than anthrax LF, that are physiological activators of NLRP1 isoforms. One possibility is that NLRP1 may be activated by self-encoded (rather than pathogen-encoded) proteases. However, the signature of positive selection we observe in the linker/cleavage region implies that a host-pathogen arms race is likely driving the rapid evolution of NLRP1. Unless the specificity of self-encoded proteases is also rapidly evolving, they would not be expected to drive rapid evolution of the protease target site in NLRP1. Thus we tend to favor the idea that NLRP1 variants evolved to recognize diverse pathogen-encoded proteases. However, identification of these proteases may be technically challenging since the pathogens producing them may have long been driven into extinction or, alternatively, evolved proteases with specificities that avoid cleaving NLRP1. Nevertheless, our results establish that a unified proteolytic mechanism underlies activation of NLRP1 variants in diverse species, and provide a basis for understanding the rapid evolution of this family of cytosolic immunosensors.

## Materials and Methods

### Plasmids and constructs

The B6-NLRP1B allele was amplified by PCR from a plasmid with a cDNA sequence representing BC141354 (Thermo Fisher Scientific Biosciences) using primers 1+2 (S4 Table), and sub-cloned into CMSCV-IRES-hCD4 using XhoI and NotI sites. For the N-terminal GFP fusion, B6-NLRP1B was amplified using primers 3+2, and subcloned into MSCV-GFP-MCS into the NotI site. A TEV site was added to the B6 allele using QuikChange Mutagenesis as described in [16] with primers 4+5. The 129 allele LF cleavage site was mutated with primers 6+7, 8+9, or 12+13 to generate 129/B6-A, 129/B6-B and K38A/K44A respectively. Then 129/B6-A was sequentially mutated with primers 10–11 to generate 129/B6-C.

An NLRP1A cDNA template was obtained from Source BioScience (BC156396.1). This sequence was amplified and cloned with primers 18+19 into pcDNA3.1-Myc-HisA using the BamHI and NotI sites. This original sequence contains three splicing differences when compared to the reference sequence NM_001004142. Two of these differences result in inclusion of two extra exons not found in the reference sequence. These exons were sequentially deleted using primer pair 22+23, followed by primer pair 24+25. The third difference was a missing 3’ exon. To correct this difference, we used SOE PCR [51] to insert the missing exon. PCR Fragment 1 was amplified with primers 29+26, and PCR fragment 2 was amplified with primers 27+19. PCR Fragment 2 was extended at its 5’ end with primers 28+19 to form fragment 3. Fragments 1 and 3 were mixed, allowed to anneal to each other, and extended and amplified with primer 29+19 to form fragment 4. Fragment 4 was digested with EcoRI and NotI, and was used to replace the EcoRI and NotI fragment released from the original NLRP1A-pCDNA that had been already corrected for the first two exons. The final NLRP1A resembling the reference sequence was amplified with primers 20+21. The 5’end of this fragment was extended with primers 17+21, and then sub-cloned into MSCV-GFP-MCS into the NotI site. A TEV-site was inserted by Quikchange using primers 30+31.

A human NLRP1 cDNA template was obtained from the American Type Culture Collection (ATCC) (I.M.A.G.E. Clone ID: 5756099), which contains a sequence resembling transcriptional variant 5 (NM_001033053.2). This cDNA is missing the last two 3’ exons that are found in transcript variant 1 (NM_033004.3) and are predicted to encode the CARD, a domain necessary for signaling. We modified the sequence of transcript variant 5 to add these last two exons to match variant 1. The last exon was amplified by PCR with primers 40+33 and gDNA from THP-1 cells (ATCC) to form fragment 1. The N-terminal fragment 2 was amplified with primers 39+32. Fragments 1 and 2 were mixed, annealed, extended, and then amplified with primer 32+33. The final PCR product was cloned into pcDNA3.1-Myc-HisA with KpnI and XhoI. The entire length of the newly modified ORF was sequenced, and a point mutation was identified and modified with primers 34+35 to resemble the reference sequence. A ΔPYD variant was amplified with primers 38+33 and cloned back into pcDNA3.1-Myc-HisA. The LRR was deleted, TEV-sites were inserted, and Walker A mutations were modified via Quikchange using primers 36+37, 41+42, 43+44, 45+46, and 47+48 respectively. GFP N-terminal fusions were constructed by amplifying hNLPR1 with primers 49, 50, 51, and 52 at the 5’end and primer 53 at the 3’end, and these fragments were cloned MSCV-GFP-MCS at the NotI site.

A human *ASC* expression plasmid was obtain through the Addgene repository (plasmid #41553) [52]. A human *IL1B* ORF was amplified from cDNA made from a human microglial cell line stimulated with LPS (gift from Kaoru Saijo at UC Berkeley) using primers 60+61. The amplified DNA was cloned into pcDNA3.1-V5. A plasmid encoding human *CASP1* (NM_033292.3) was obtained from the Dietrich Lab (Harvard Medical School).

The NS2B/NS3 coding sequence from Dengue virus type 3 isolate D3/H/IMTSSA-MART/1999/1243 was synthesized (IDT) with 5’ XhoI and 3’ NotI sites for sub-cloning into the plasmid pQCXIP, resulting in the addition of a 5’ HA tag.

### Evolutionary analyses

Full-length primate NLRP1 gene sequences were collected from public databases (see S1 Table for accession numbers). To augment the publicly available NLRP1 sequences, we amplified and sequenced the 5' region of NLRP1 (corresponding to the linker region between the PYD and NBD) from RNA isolated from three additional primate cell lines (Coriell Institute for Medical Research) (Mandrill: PR00399, saki monkey: PR00239 and howler monkey: PR00708) by RT-PCR using primers pNLRP1fwd pNLRP1rev shown in S4 Table. These additional sequences have been deposited to Genbank (S1 Table). All sequences were aligned based on their translated sequence and alignments were manually curated in Geneious [53]. Phylogenetic trees were generated using PhyML [54]. Maximum likelihood evolutionary analyses for positive selection were performed using PAML [46] or the PARRIS [47] package implemented at datamonkey.org (http://www.datamonkey.org/). Reported p-values compare the log likelihood values for models that disallow or allow for codons to evolve under positive selection. Specific codons that have evolved under recurrent positive selection with a posterior probability of >0.90 were identified using PAML [46] or the FUBAR [55] package implemented at datamonkey.org. Sliding window analyses were performed using K-estimator [56].

### Cell culture and transfection and lethal toxin

HEK 293T (ATCC) cells were grown in complete media (DMEM, 10% FBS, 100 U/ml Penicillin, 100 μg/ml Streptomycin, and supplemented with 2mM L-glutamine). HEK 293T cells were seeded the day prior to transfection at a density of 1.5x10^5^ cells/well in a 24-well plate with complete media. DNA complexes were made with Lipofectamine 2000 (Invitrogen) according to manufacturer’s instructions and overlaid on cells for 24–36 hours prior to analysis. Amounts of transfected DNA were normalized with empty vector when necessary. Lethal toxin (LeTx), comprised of recombinant *E*. *coli*-expressed His-tagged lethal factor (LF) and His-tagged protective antigen (PA), was the kind gift of Bryan Krantz [57]. In some experiments, instead of exposing cells to LeTx protein, cells were instead transfected with a cDNA for LF, as indicated in the figure legends.

### Immunoblotting

Cells were lysed in RIPA buffer supplemented with 1mM PMSF and 1× Complete Protease Inhibitor Cocktail (Roche). Lysates were spun at max speed in an Eppendorf microfuge at 4°C for 20 min and supernatants were mixed with 6× Laemmli sample buffer. To detect full length and FIIND processed NLRP1B, lysates were incubated at room temperature for 15min prior to SDS-PAGE. To analyze all other proteins, including the N-terminally cleaved form of NLRP1B, samples were boiled for 10min prior to separation. SDS-PAGE was performed with Novex 10% and 12% BisTris gel system according to manufacturer’s instructions (Invitrogen). Separated proteins were transferred to Immobilon-FL PVDF membranes. Membranes were blocked with Odyssey blocking buffer (Licor). The following antibodies were used for the following antigens: HA mAB 3F10 (Roche), MEK-2 SC-13115 (Santa Cruz), MYC mAb 9E10 (Clonetech), IL-1β AF-401-NA (R&D systems). Secondary antibodies anti-rat, mouse and goat were all conjugated to Alexa Fluor-680 (Invitrogen).

## Supporting Information

S1 Fig Activation of the human NLRP1 inflammasome by proteolysis is protease-specific. 293T cells were co-transfected with plasmids encoding mouse CASP1 (100ng), IL1b (100ng) and either 100ng EGFP-HA-NLRP1B-129 or EGFP-HA-TEV-NLRP1B-129, and either 400ng lethal factor protease (pLF), NS2B/NS3 protease (pNS), TEV protease (pTEV) or empty vector (V). Non-boiled lysates were subject to immunoblot (IB) with antibodies specific for IL-1β to detect CASP1-dependent processing of IL-1β into the mature p17 fragment (A), or HA to detect the N-terminus of NLRP1B (upper panel) or self-cleavage of NS2B/NS3 (lower panel) (B).
(TIF)

S2 Fig Mutational analysis of the LF cleavage site in the 129 NLRP1B isoform. (**A**) Alignment of the amino acid sequences of the B6 and 129 alleles of mouse NLRP1B in the vicinity of the LF cleavage site (in the 129 variant). Numbering is based on the 129 allele. The LF cleavage site (between K44 and L45 of the 129 allele) is indicated by an arrowhead. Sequences of 129-based constructs mutated to resemble the B6 sequence are also shown. (**B, C**) 293T cells were transfected with 200ng and 800ng of GFP-HA-NLRP1B constructs for 24h, and then treated with anthrax lethal toxin (LeTx, 1μg/ml) overnight. In (B) cells were co-transfected with 200ng each of expression constructs for CASP1 and IL-1β. Non-boiled lysates were subject to immunoblot (IB) with anti-HA antibody, whereas boiled lysates were subject to immunoblot with anti-IL-1β to detect CASP1-dependent processing of IL-1β into the mature p17 fragment.
(TIF)

S3 Fig Evolutionary analysis of mammalian NLRP1 homologs. (**A**) Schematic of the chromosomal arrangement of several mammalian NLRP1 homologs showing that synteny has been conserved across a broad range of mammalian evolution. Mice are distinct from the other shown lineages by having two NLRP1 paralogs (NLRP1A and NLRP1B) as well as the fact that the mouse proteins lack the N-terminal Pyrin domain (shown as an empty dashed box) that is found in many other mammalian lineages. (**B**) A phylogenetic tree of NLRP1 proteins shown in part A. (**C**) Pairwise sliding window comparison of the dN/dS ratio between mouse NLRP1 paralogs, NLRP1A and NLRP1B. dN/dS ratios were calculated every 20 codons with a window size of 50 codons. Shown below is the domain structure of mouse NLRP1B.
(TIF)

S4 Fig Secondary structure prediction of human NLRP1. Secondary structure of the first N-terminal 320 residues of human NLRP1 were predicted with PSIPRED. Alpha helices are depicted as pink cylinders and beta-sheets as yellow arrows. The location of engineered TEV cleavage-sites are indicated with red arrows above the primary sequence.
(TIF)

S1 Table Primate NLRP1 accession numbers. (PDF)

S2 Table Positively selected amino acid positions based on sequences of the NLRP1 linker region from 20 primates. (PDF)

S3 Table Positively selected amino acid positions based on whole gene sequences of NLRP1 from 11 primates. (PDF)

S4 Table Oligonucleotide sequences used for PCR amplification or site directed mutagenesis. (PDF)
