# Supplementary figures and images for: Functional and Evolutionary Analyses Identify Proteolysis as a General Mechanism for NLRP1 Inflammasome Activation
Source: PLoS Pathog. 2016 Dec 7;12(12):e1006052. doi: 10.1371/journal.ppat.1006052 (PMC5142783; doi:10.1371/journal.ppat.1006052)

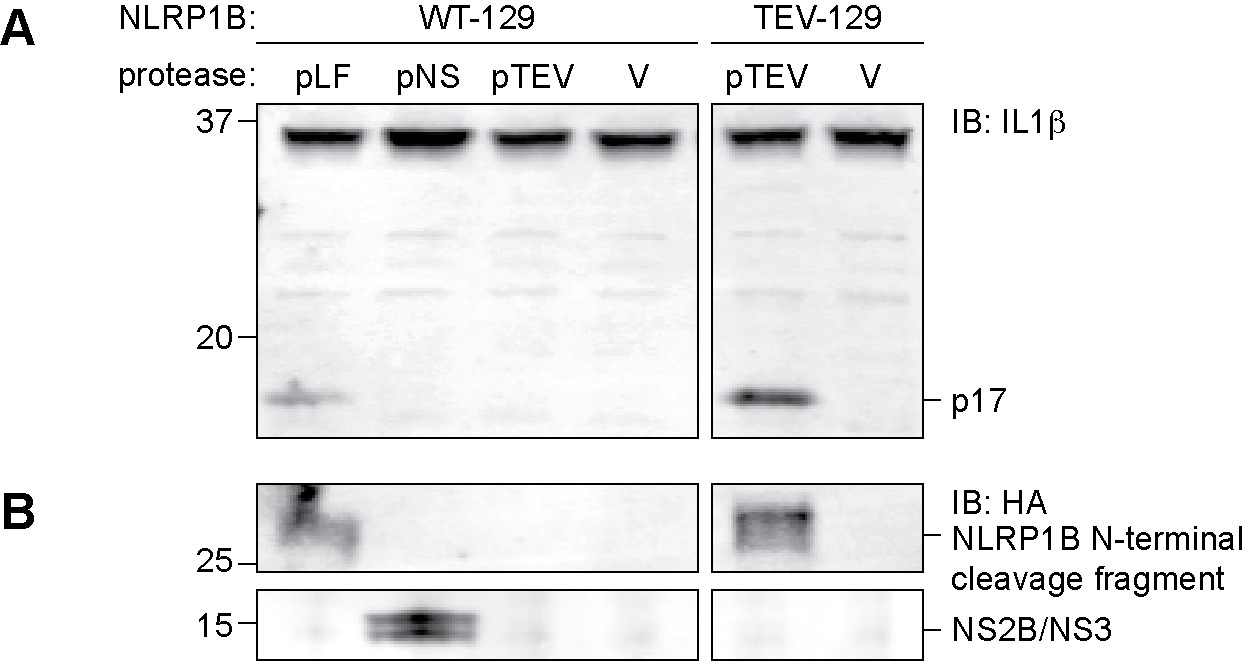

Supplement: S1 Fig — 293T cells were co-transfected with plasmids encoding mouse CASP1 (100ng), IL1b (100ng) and either 100ng EGFP-HA-NLRP1B-129 or EGFP-HA-TEV-NLRP1B-129, and either 400ng lethal factor protease (pLF), NS2B/NS3 protease (pNS), TEV protease (pTEV) or empty vector (V). Non-boiled lysates were subject to immunoblot (IB) with antibodies specific for IL-1β to detect CASP1-dependent processing of IL-1β into the mature p17 fragment (A), or HA to detect the N-terminus of NLRP1B (upper panel) or self-cleavage of NS2B/NS3 (lower panel) (B). (TIF) [file ppat.1006052.s001.tif]

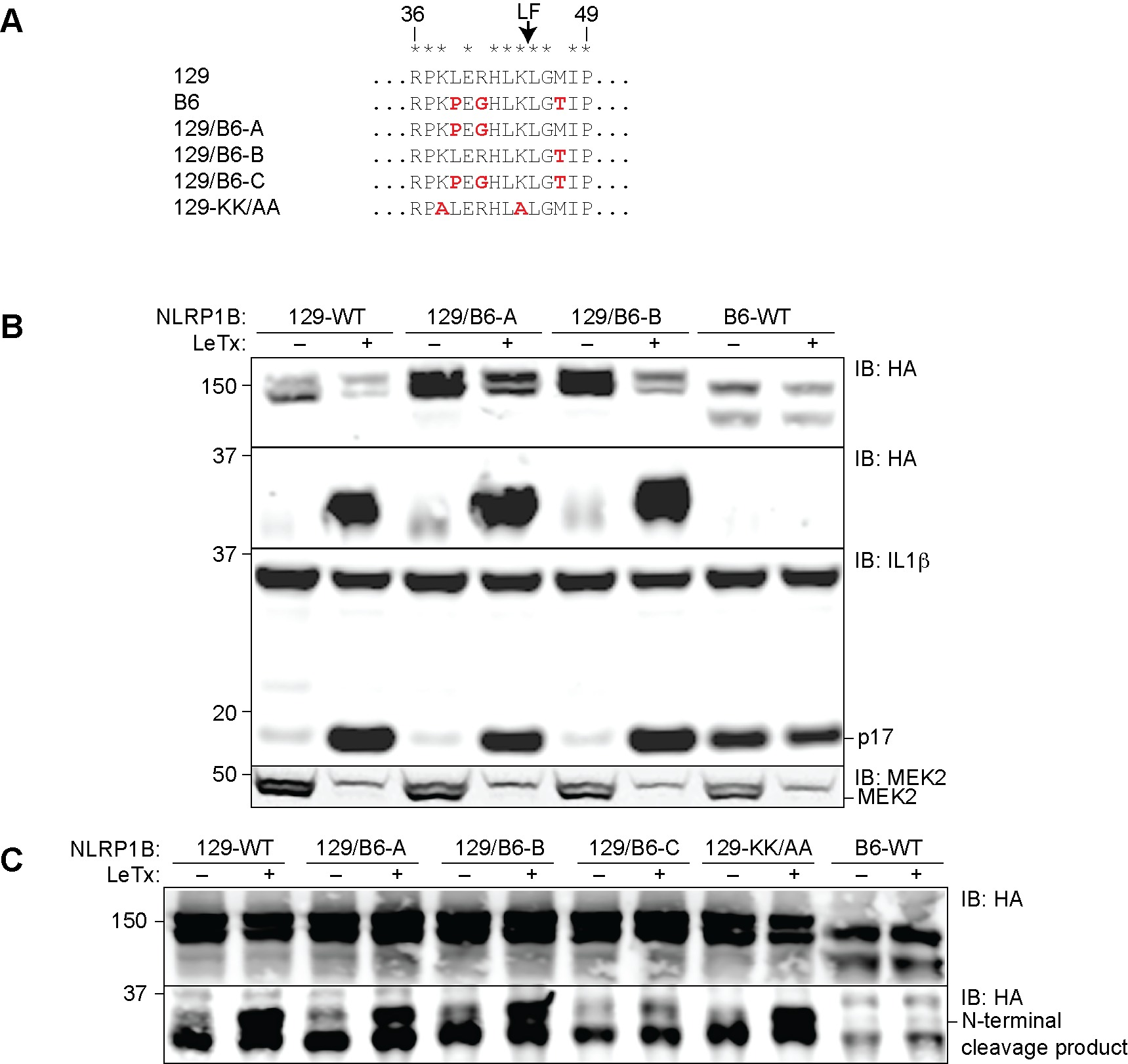

Supplement: S2 Fig — (A) Alignment of the amino acid sequences of the B6 and 129 alleles of mouse NLRP1B in the vicinity of the LF cleavage site (in the 129 variant). Numbering is based on the 129 allele. The LF cleavage site (between K44 and L45 of the 129 allele) is indicated by an arrowhead. Sequences of 129-based constructs mutated to resemble the B6 sequence are also shown. (B, C) 293T cells were transfected with 200ng and 800ng of GFP-HA-NLRP1B constructs for 24h, and then treated with anthrax lethal toxin (LeTx, 1μg/ml) overnight. In (B) cells were co-transfected with 200ng each of expression constructs for CASP1 and IL-1β. Non-boiled lysates were subject to immunoblot (IB) with anti-HA antibody, whereas boiled lysates were subject to immunoblot with anti-IL-1β to detect CASP1-dependent processing of IL-1β into the mature p17 fragment. (TIF) [file ppat.1006052.s002.tif]

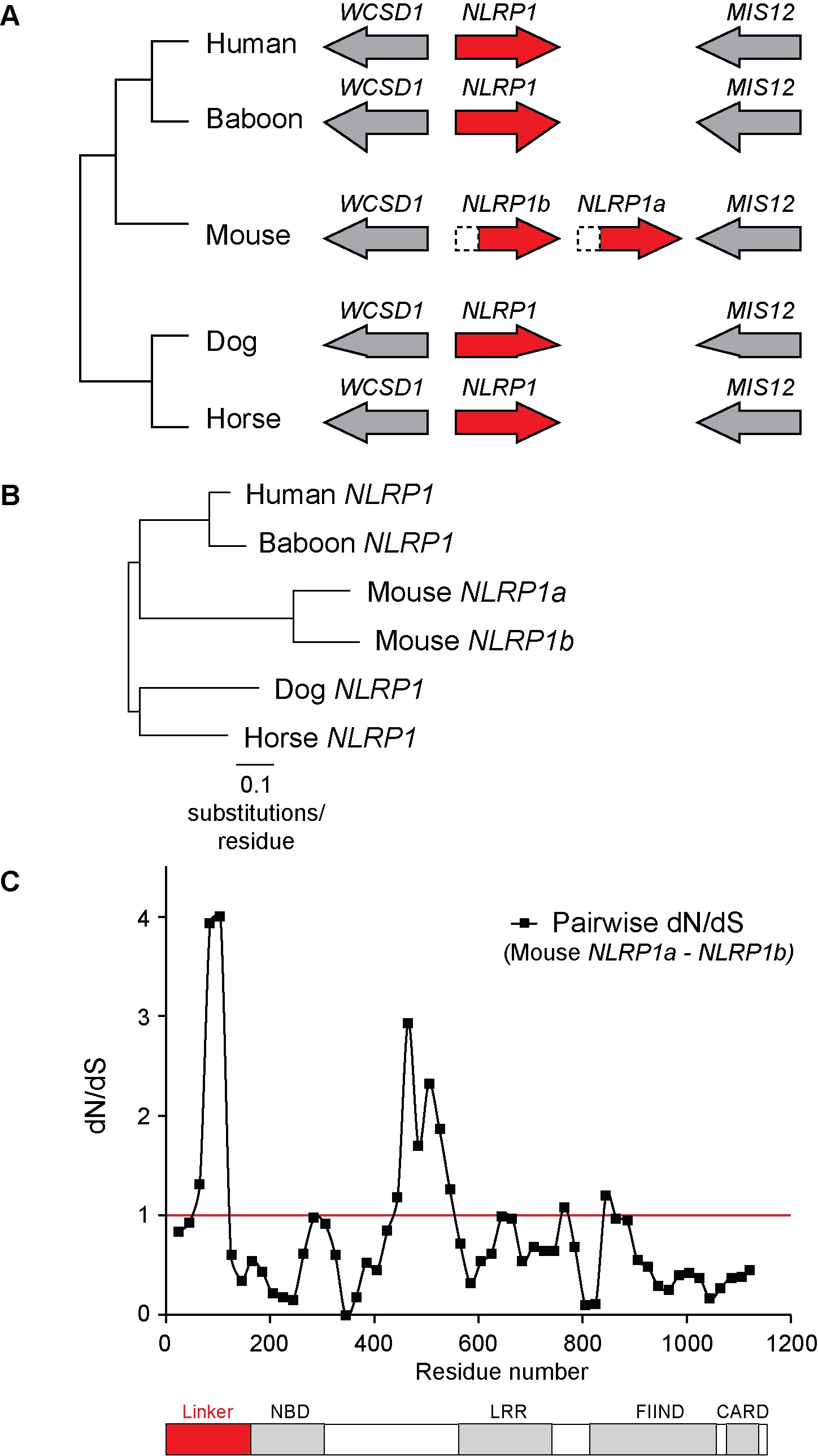

Supplement: S3 Fig — (A) Schematic of the chromosomal arrangement of several mammalian NLRP1 homologs showing that synteny has been conserved across a broad range of mammalian evolution. Mice are distinct from the other shown lineages by having two NLRP1 paralogs (NLRP1A and NLRP1B) as well as the fact that the mouse proteins lack the N-terminal Pyrin domain (shown as an empty dashed box) that is found in many other mammalian lineages. (B) A phylogenetic tree of NLRP1 proteins shown in part A. (C) Pairwise sliding window comparison of the dN/dS ratio between mouse NLRP1 paralogs, NLRP1A and NLRP1B. dN/dS ratios were calculated every 20 codons with a window size of 50 codons. Shown below is the domain structure of mouse NLRP1B. (TIF) [file ppat.1006052.s003.tif]

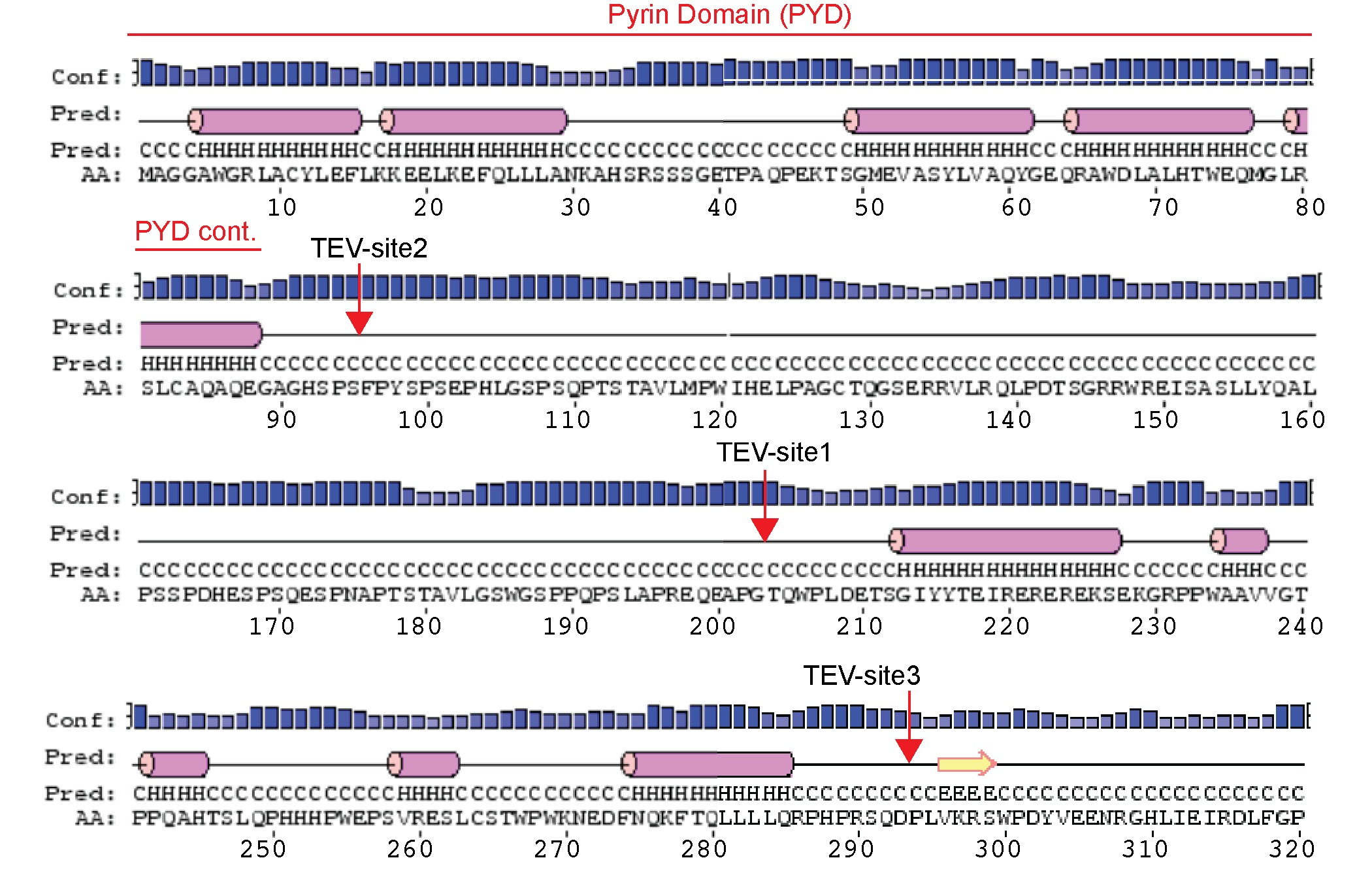

Supplement: S4 Fig — Secondary structure of the first N-terminal 320 residues of human NLRP1 were predicted with PSIPRED. Alpha helices are depicted as pink cylinders and beta-sheets as yellow arrows. The location of engineered TEV cleavage-sites are indicated with red arrows above the primary sequence. (TIF) [file ppat.1006052.s004.tif]
