# Supplementary material for: Functional and Evolutionary Analyses Identify Proteolysis as a General Mechanism for NLRP1 Inflammasome Activation
Source: PLoS Pathog. 2016 Dec 7;12(12):e1006052. doi: 10.1371/journal.ppat.1006052 (PMC5142783; doi:10.1371/journal.ppat.1006052)
Supplement: S1 Table — (PDF) [file ppat.1006052.s005.pdf]

Table S1. Primate NLRP1 accession numbers

Species	Accession number
Human (full-length)	NM_033004.3
Chimpanzee (full-length)	XM_009431692.1
Gorilla (linker)	XM_004058386.1
Orangutan (full-length)	XM_002826912.3
White-cheeked gibbon (full-length)	XM_012501543.1
Mandrill (linker)	KX579894
Drill (full-length)	XM_011994673.1
Baboon (linker)	XM_009189476.1
Sooty mangabey (full-length)	XM_012054373.1
Pig-tailed macaque (linker)	XM_011729025.1
Crab-eating macaque (linker)	XM_005595922.2
Rhesus macaque (full-length)	NM_001114349.1
African green monkey (linker)	XM_008010035.1
Snub-nosed monkey (full-length)	XM_010355833.1
Colobus (linker)	XM_011958625.1
Marmoset (full-length)	XM_008997299.1
Owl monkey (full-length)	XM_012460520.1
Squirrel monkey (full-length)	XM_010341964.1
Howler monkey (linker)	KX579892
Saki monkey (linker)	KX579893
