# Supplementary material for: Functional and Evolutionary Analyses Identify Proteolysis as a General Mechanism for NLRP1 Inflammasome Activation
Source: PLoS Pathog. 2016 Dec 7;12(12):e1006052. doi: 10.1371/journal.ppat.1006052 (PMC5142783; doi:10.1371/journal.ppat.1006052)
Supplement: S2 Table — (PDF) [file ppat.1006052.s006.pdf]

Table S2. Positively selected amino acid positions based on sequences of the NLRP1 linker region from 20 primates

Region	Residue Number (from human NLRP1)	Posterior probability PAML	FUBAR
Linker	105	0.951	
Linker	120	0.985	
Linker	122	0.978	0.978
Linker	139		0.906
Linker	146		0.943
Linker	166	0.975	0.962
Linker	171	0.921	0.910
Linker	187	0.939	0.910
Linker	211	0.997	0.940
Linker	292	0.918	
Linker	293	0.961	0.961
Linker	298	0.975	0.974
