# Supplementary material for: Functional and Evolutionary Analyses Identify Proteolysis as a General Mechanism for NLRP1 Inflammasome Activation
Source: PLoS Pathog. 2016 Dec 7;12(12):e1006052. doi: 10.1371/journal.ppat.1006052 (PMC5142783; doi:10.1371/journal.ppat.1006052)
Supplement: S3 Table — (PDF) [file ppat.1006052.s007.pdf]

Table S3. Positively selected amino acid positions based on whole gene sequences of NLRP1 from 11 primates

Region	Residue Number (from human NLRP1)	Posterior probability PAML	Posterior probability FUBAR
Linker	105	0.963	
Linker	120	0.938	
Linker	122	0.931	
Linker	166	0.927	
Linker	211	0.991	
Linker	293	0.956	0.910
	501	0.940	
	569	0.985	
	652	0.918	
	680	0.902	
	729	0.974	
	762	0.966	0.910
LRR	834	0.910	
LRR	891	0.991	0.957
LRR	893	0.974	
LRR	912	0.917	
LRR	923	0.972	
LRR	937	0.904	
LRR	961	0.918	
	991	0.987	0.963
	995	0.976	0.927
	1016	0.971	0.937
	1052	0.979	0.954
	1059	0.951	
	1356	0.975	
	1366	0.964	0.939
CARD	1398	0.961	0.913
CARD	1443	0.939	0.934
