# Supplementary material for: Functional and Evolutionary Analyses Identify Proteolysis as a General Mechanism for NLRP1 Inflammasome Activation
Source: PLoS Pathog. 2016 Dec 7;12(12):e1006052. doi: 10.1371/journal.ppat.1006052 (PMC5142783; doi:10.1371/journal.ppat.1006052)
Supplement: S4 Table — (PDF) [file ppat.1006052.s008.pdf]

Table S4 Oligonucleotide sequences used for PCR amplification or site directed mutagenesis.

NLRP1B	Sequence	Short oligo name
1	tataCTCGAGccacc ATG TACCCATACGACGTCCCAGACTACGCT GAACAATCTCAGCCC	B6 NLRP1-XhoI-HA
2	atatGCGGCCGC TCA GGATCCCAAAGAGACCC	B6 NLRP1-R-stop-NotI
3	atatGCGGCCGCt ATGTACCCATACGACGTCC	GFP-HA-F-NotI
4	GCAGACCCAAG GAGAACCTGTATTTTCAGGGC GGGACAATTCCAAAAGTACACATAAAAC	B6-N1b-TEV3-F
5	CTTTTGGAATTGTCCCgccctgaaaatacaggttctcCTTGGGTCTGCGCTTCATCAGC	B6-N1b-TEV3-R
6	AGAGCTGAAGCACAGACCCAAG ccc GAG ggg CACTTGAAGCTAGGAATGATTCCAGTAG	129-B6-A-F
7	GGAATCATTCCTAGCTTCAAGTGcccCTCgggCTTGGGTCTGTGCTTCAGCTCTACATTC	129-B6-A-R
8	CTAGAGAGACACTTGAAGCTAGGA acc ATTCCAGTAGTATATATGAAGCAGGGAGAAGAG	129-B6-B-F
9	CTCCCTGCTTCATATATACTACTGGAATggtTCCTAGCTTCAAGTGTCTCTCTAGCTTGG	129-B6-B-R
10	CCCGAGGGGCACTTGAAGCTAGGA acc ATTCCAGTAGTATATATGAAGCAGGGAGAAGAG	129-B6-C-F
11	CTCCCTGCTTCATATATACTACTGGAATggtTCCTAGCTTCAAGTGCCCCTCGGGCTTGG	129-B6-C-R
12	GACCCgcGCTAGAGAGACACTTG gcG CTAGGAATGATTCCAGTAGTATATATGAAGCAGG	N1b-K38A-K44A-F
13	TATATACTACTGGAATCATTCCTAGCgcCAAGTGTCTCTCTAGCgcGGGTCTGTGCTTC	N1b-K38A-K44A-R
14	atatCTCGAGgccaccATG CTAGAGAGACACTTGAAGCTAGG	N1b-L39-XhoI-F
15	atatCTCGAGgccaccATG CTA GGAATGATTCCAGTAGTATATATGAAGC	L45-XhoI
16	atatCTCGAGgccaccATG GGAATGATTCCAGTAGTATATATGAAGC	G46-start-xhoI
17	atatGCGGCCGCTCAAGCGTAGTCTGGGACGTCG	HA-stop-NotI-R
18	CTGCTGGGATTGGG Aga TCt ACACTGGCCAGGCTGGTG	Nlrp1b-K137R-F
19	CAGCCTGGCCAGTGTaGAtcTCCCAATCCCAGCAGCCC	Nlrp1b-K137R-R
**NLRP1A**		
20	gcatat GGATCC gccacc ATGGAAGAATCTCAGTCCAAGCAGG	NLRP1A-BamHI-F
21	gcatatGCGGCCGCccTTTCACAGAGACCCCACCCAAC	NLRP1A-NotInostop-R
22	atCTCGAGgccaccATG TACCCATACGACGTCCCAGACTACGCT GAAGAATCTCAGTCCAAGCAGG	HA-NLRP1A-XhoI-F
23	ggcatGCGGCCGC TCA GGATTTCACAGAGACCCCACC	NLRP1A-stop-NotI-R
24	GGCTGCCAACTAAAG ACT CTG TGG CTTGTTGAATGCGGCCTCACATCCACATAC	NLRP1A-exonAdel-F
25	ATGTGAGGCCGCATTCAACAAGCCACAGAGTCTTTAGTTGGCAGCCACGC	NLRP1A-exonAdel-R
26	TGAAGCAGCAGAGACAGCAGTCA GGAGACAAACACATGGAACCTCTGGGG	NLRP1A-exonBdel-F
27	GAGGTTCCATGTGTTTGTCTCCTGACTGCTGTCTCTGCTGCTTCAGTAGG	NLRP1A-exonBdel-R
28	GGT GATCTCAGACCTGCACTGC CCAAGATTGCTACAGCCCCC	NLRP1A-SOE
29	GGGGGCTGTAGCAATCTTGGGCAGTGCAGGTCTGAGATCACC	NLRP1A-SOE
30	GCAGTGCAGGTCTGAGATCACCTGGTTTCAGCAAGGCCTCCC	NLRP1A-C' ext3
31	gcagtgacaatagagatcgaattctgtgcc	Nalp1b Seq4
32	TGAAGCACAGAGTgCAG gag AAC CTG TAT TTT CAG ggc AGAACCTTTCCAGGAGCACGTAC	NP1A-TEV3-F2
33	TCCTGGAAAGGTTCTgccCTGAAAATACAGGTTctcCTGcACTCTGTGCTTCATTAGCTCC	NP1A-TEV3-R2
**hNLRP1**		
34	gctataGGTACC gccacc ATGGCTGGCGGAGCC	hNLRP1-F-KpnI-Kzk
35	gctata CTCGAG GCTGCTGAGTGGCAGGAG	hNLRP1-R-noStop-XhoI
36	GCTGCTACTTCTACAAAGACCTCACCCCAGAAGCCAAGATCCCCTGG	hNP1 C1414T-F
37	GGGATCTTGGCTTCTGGGGTGAGGTCTTTGTAGAAGTAGCAGCTGTGTG	hNP1 C1414T-R
38	CCTCAAGGTCACCAGAAACCCTCAGCTGCTCATCTTCAGC	hNP1-dLRR2-F
39	GAAGATGAGCAGCTGAGGGTTTCTGGTGACCTTGAGGACG	hNP1-dLRR2-R
40	gctata GGTACC gccaccATG ACCCAATGGCCTCTGG	hNP1-PC1
41	CGGGCTGGAGGGATCAGAGTAGTTGCAGGCATGAGATCTCCTGGTTTCACCAAGGCCTCC	hNP1-R-Nt-SOE
42	CTACTCTGATCCCTCCAGCCCG CATAG CCGTACCTTCACCTCTGGATGCC	hNPR1-F-Ct-SOE
43	GGGCTGCTGGAATTGGG gcG TCAACACTGGCCAGGCAGGTGAAGGAAG	hNLRP1-WalkerA-K-A-F

44	CTTCACCTGCCTGGCCAGTGTTGACgcCCCAATTCCAGCAGCCCCCTGC	hNLRP1-WalkerA-K-A-R
45	GGGCTGCTGGAATTGGG agG TCAACACTGGCCAGGCAGGTGAAGGAAG	hNLRP1-WalkerA-K-R-F
46	CTTCACCTGCCTGGCCAGTGTTGAcctCCCAATTCCAGCAGCCCCCTGC	hNLRP1-WalkerA-K-R-R
47	GCGCCCAAGCCCAGGAA AACCTGTATTTTCAGGGC TCATTCCCCTACAGCCCAAGTG	hNP1 TEV2-F
48	GGGCTGTAGGGGAATGAGCCCTGAAAATACAGGTTTTCCTGGGCTTGGGCGCACAGTG	hNP1- TEV2 R
49	CCTAGCACCCAGA GAG aac ctg tat ttt cag ggc CAATGGCCTCTGGATGAAACG	hNP1-Tev1-F
50	CCAGAGGCCATTG gcc ctg aaa ata cag gtt CTCTCTGGGTGCTAGGCTGG	hNP1-TEV1-R
51	atatGCGGCCGCt ATGGCTGGCGGAGCCTGGGG	GFP-notI FL-hNP1
52	atatGCGGCCGCt CCCTCATTCCCCTACAGCCC	GFP-notI-dPYD
53	atatGCGGCCGCt GAA AACCTGTATTTTCAGGGC TCATTCC	GFP-notI-dPYD-T2
54	atatGCGGCCGCt GAG aac ctg tat ttt cag ggc CAA	GFP-notI-d198-T1
55	tgcgat GTCGAC TTAATTCAGATCCTCTTCTGAGATGAGT	MYC-stop-SalI-R
56	ATCTTCCCAATTGCTGAGATTGCA GAGGAAAGCTCCCCAGAGGTAGTACC	hNLRP1-del GKSH-F
57	TACCTCTGGGGAGCTTTCCTCTGCAATCTCAGCAATTGGGAAGATCTTGC	hNLRP1-del GKSH-R
58	CCGCCTGGCCTGTTACTTGG	pNLRP1fwd
59	GACAGGATCTGTCTAATGGGAGCC	pNLRP1rev

Other genes

60	gcatat GGATCC gccacc ATGGCAGAAGTACCTGAGCTC	hIL1b-F-BamHI
61	gcatatGCGGCCGCccGGAAGACACAAATTGCATGGTG	hI1lb-R-nostop-NotI
